# Conserved role of medium acidification in chronological senescence of yeast and mammalian cells

**DOI:** 10.18632/aging.100412

**Published:** 2011-12-17

**Authors:** Paola Fabrizio, Min Wei

**Affiliations:** ^1^ Laboratoire de Biologie Moléculaire de la Cellule, Centre National de la Recherche Scientifique, Ecole Normale Supérieure de Lyon, Université de Lyon, Lyon, France; ^2^ Andrus Gerontology Center, Dept. of Biological Sciences and Norris Cancer Center, University of Southern California, Los Angeles, CA 90089-0191, USA

The yeast chronological life span (CLS) model has led to the identification of the pro-aging effects of the TOR-Sch9 /S6K and Ras-Adenylate cyclase-PKA pathways, components of which play conserved role in nutrient sensing and aging in mammals [1-4]. One of the early changes that occurs in yeast cells grown in media containing 2% glucose and excess amino acids is the production of acetic acid and acidification of the medium to below pH 4. This acidification has been shown to accelerate yeast aging [5-9]. However, it is clear that it does not explain the effect of the TOR-Sch9/S6K and Ras-AC-PKA pathways on aging since their inhibition extends chronological life span in media that is not acidified and that does not contain acetic acid [10]. The assumption that acetic acid is an organic toxin, which is the key mediator of chronological aging under standard conditions, is probably not true for most genetic backgrounds, since under physiological conditions acetic acid is generated at low levels compared to another metabolite, ethanol [6-7, 11]. Additionally, acetic acid, in spite of its potential toxicity, represents one among several carbon sources that can be utilized by Saccharomyces cerevisiae for growth and metabolism [12-15].

In previous issue of Aging, Leontieva and Blagosklonny describe a yeast-like chronological senescence (CS) model in mammalian cells (Leontieva and Blagosklonny). They show that human tumor cells maintained in stationary culture lose their viability (colony forming units) and that this process is accelerated by medium acidification caused in part by lactate accumulation, which mirrors the accumulation of ethanol and some acetic acid, and the acidification of the medium in S. cerevisiae [5-7, 9]. In yeast, the ethanol accumulated during the growth phase can be used as carbon source during the diauxic shift and the post-diauxic phase, when cells stop dividing and switch from a fermentation- to a respiration-based metabolism [5, 16-17]. Long-lived mutants with deficiencies in the TOR- Sch9/S6K and Ras-AC-PKA pathways deplete ethanol, show a reduced accumulation of extracellular acetic acid [6, 11](M. Wei unpublished results) as well as activate glycerol biosynthesis [11]. As opposed to glucose and ethanol and, possibly, acetic acid, glycerol does not elicit adverse effects on cellular protection and life span suggesting that the Tor1/Sch9-regulated glycerol biosynthesis results in the removal of pro-aging carbon sources [11].

Leontieva and Blagosklonny show that the “yeast-like” chronological senescence in mammalian cells is delayed and attenuated by the inhibition of the mTOR and PI3K signaling pathways, both of which have been implicated in longevity regulation in organisms ranging from yeast to mice. Conditioned medium produced by rapamycin-treated cells was less toxic in inducing CS. However, the addition of rapamycin did not protect fibrosarcoma cells from high concentration of lactate suggesting that rapamycin did not protect cells from CS per se. Rather, inhibition of mTOR affected cellular metabolism and inhibited lactate production during the early phase of stationary survival, which led to a reduced initial lactate accumulation and delayed CS (Leontieva and Blagosklonny). Interestingly, mTOR was spontaneously inactivated after one day in culture, possibly a protective response to lactate accumulation and medium acidification. These results suggest that mTOR promotes CS by favoring lactate production and medium acidification in agreement with the role for TOR-Sch9/S6K in promoting ethanol and acetic acid accumulation in yeast [5, 11, 18]. By contrast, the deletion of either TOR1 or SCH9/S6K are known to extend yeast chronological life span in part by depleting ethanol and acetic acid but largely by mechanisms that are cell autonomous [10-11, 19-21].

It has been argued that acidification of the culture medium and the accumulation of non-fermentable carbon sources such as ethanol and acetic acid render the CLS a paradigm for the identification of “private” mechanisms specific for yeast chronological aging [7, 22-23]. However, not only the yeast CLS method has been remarkably effective in discovering genes later shown to promote aging in mammals [4], it has also revealed the multi-factorial nature of yeast chronological senescence and points to the involvement of diverse cellular processes, such as mitochondrial respiration, reactive oxygen species signaling [1, 19, 24-27], stress response [3, 10, 28], autophagy [29-30], and genome maintenance, in the regulation of life span [31-35]. Although, accumulation of toxic metabolic byproducts may not represent a mechanism of aging in yeast [5-8] or mammalian cells (Leontieva and Blagosklonny [36]), chronological senescence provides a simple model for probing the roles of genes and signaling pathways that affect aging and a powerful platform for high-throughput screening of agents that modulate aging and age-related disease progression.
